# Dataset on energy efficiency assessment and measurement method for child-friendly space in cold residential area

**DOI:** 10.1016/j.dib.2017.07.032

**Published:** 2017-07-19

**Authors:** Kai Ren, Leiqing Xu

**Affiliations:** a Water Conservancy and Civil Engineering College, Inner Mongolia Agriculture University, Zhaowuda Road No.306, Hohhot, Inner Mongolia, China; b College of Architecture and Urban Planning, Tongji University, Siping Road No.1239, Shanghai, China

**Keywords:** Cold residential area, Children friendly space, Environment behavior, Energy efficiency assessment, Measurement method

## Abstract

The data related in this paper are related to “Environmental-behavior studies of sustainable construction of the third place – based on outdoor environment-behavior cross-feed symbiotic analysis and verification of selective activities” (Ren, 2017) [1]. The dataset was from a field sub-time extended investigation to children of Hohhot West Inner Mongolia Electric Power Community Residential Area in Inner Mongolia of China that belongs to cold region of ID area according to Chinese design code for buildings. This filed data provided descriptive statistics about outdoor time, behavior scale specificity, age exclusivity and self-centeredness for children in different ages (babies, preschool children, school age children). This data provided five measurement elements of child-friendly space and their weight ratio. The field data set is made publicly available to enable critical or extended analyzes.

## Specifications Table

**Table:** 

**Subject area**	Environment, Space, Behavior
**More specific subject area**	Children' outdoor behaviors in cold climate and children friendly space.
**Type of data**	Tables, Text file
**How data was acquired**	Outdoor-time and behavior-system were observed by field sub-time extended investigation to children; Cognition of child-friendly space and its weight ratio were observed by questionnaire, researching, observing and mathematical statistics.
**Data format**	Raw, Analyzed
**Experimental factors**	Publicly available data sources in all seasons.
**Experimental features**	Relationship between children’ outdoor behaviors and outside space (environment) was assumed continuous interacted, and assessment and measurement method of space were given.
**Data source location**	Hohhot, Inner Mongolia, China, 40°29′28.01′′ N, 111°47′07.69′′ E
**Data accessibility**	The data are available within this article.

## Value of data

• The data presents children' activities of different ages in cold area communities such as outdoor time, behavior scale, comprehensive exclusivity and self-centered. The data also provides the proportion and measuring contents of the five criteria (seasonal circulation, accessibility, safety, versatility and comfort) of child-friendly public space in cold residential areas.
• The data presents the assessment and measurement method of child-friendly public space in cold residential areas.
• Other researchers may find the data useful for different types of analysis in areas such as urban public planning and child-healthy community.
• The data are publicly available, but are dispersed within several different sources.

## 1 Data

The dataset of this article provides information on children' activities and outdoor time of different ages in cold area communities (babies, preschool children, school age children) [2], [3], and it provides the proportion and measuring contents of child-friendly public space in cold residential areas. Table 1 shows various stages of life circle and their percentage. Table 2, Table 3, Table 4 shows outdoor time distribution of children and their percentage. Table 5, Table 6, Table 7 shows other cognitions of friendly space in children of different ages. Table 8, Table 9 shows measurement elements and spatial form of child-friendly space.

**Table 1:** Various stages of life circle and their percentage.

**Stage**	**Age segmentation (years old)**	**Percentage in total population**
**Baby and school children**	0–14	26
**Unmarried young people**	15–24	12
**Married young, no children**	15–34	3
**Married young, having children**	15–34	12
**Married middle-aged, having children**	35–44	11
**Married aged, having children**	45–64	5
**Married aged, no children**	45–64	18
**Old man who relies on the pension**	More than 64	13

**Table 2:** Outdoor time and their percentage (0–3 years old babies). (Spring and Autumn Research Time: 2017.4.1–2017.4.3. Research Location: Hohhot West Mongolia Electric Power Community. Questionnaire sample:39 were issued, 36 were recovered and 27 valid questionnaires.).

**Valid period**	**Summer** **(June, July, August)**	**Spring (autumn)** **(April, May, September, October)**	**Winter** **(November, December, January, February, March)**
**8:00–9:00**	8%	5%	1%
**9:00–10:00**	15%	8%	3%
**10:00–11:00**	18%	13%	6%
**11:00–12:00**	5%	20%	15%
**12:00–13:00**	2%	5%	21%
**13:00–14:00**	1%	3%	10%
**14:00–15:00**	1%	5%	15%
**15:00–16:00**	5%	7%	11%
**16:00–17:00**	7%	10%	6%
**17:00–18:00**	12%	9%	5%
**18:00–19:00**	18%	9%	3%
**19:00–20:00**	5%	4%	2%
**20:00–21:00**	2%	1%	1%
**21:00–22:00**	1%	1%	1%

**Table 3:** Outdoor time and their percentage (3–6 years old preschool children). {For residential area that having kindergartens, children' activity time (3–6 years old) is combined with kindergarten education (outdoor time), and the research is integrated. Questionnaire sample: choose the middle class in Electric Kindergarten to do sample analysis, 30 were issued, 30 were recovered and 28 valid questionnaires.}.

**Valid period**	**Summer** **(June, July, August)**	**Spring(autumn)** **(April, May, September, October)**	**Winter** **(November, December, January, February, March)**
**8:00–9:00**	10%	7%	5%
**9:00–10:00**	15%	12%	4%
**10:00–11:00**	2%	10%	6%
**11:00-12:00**	1%	1%	22%
**12:00–13:00**	1%	1%	1%
**13:00–14:00**	1%	1%	1%
**14:00–15:00**	1%	10%	12%
**15:00–16:00**	5%	15%	18%
**16:00–17:00**	15%	10%	6%
**17:00–18:00**	25%	21%	16%
**18:00–19:00**	16%	6%	5%
**19:00–20:00**	5%	3%	2%
**20:00–21:00**	2%	1%	1%
**21:00–22:00**	1%	1%	1%

**Table 4:** Outdoor time and their percentage (6–12 years old school age children). (The activities of school-age children in residential areas are concentrated on holidays. The survey was conducted just during the Tomb sweeping days, so the results shown were the expected results. Tracking sample: do interview and questionnaire to 11 10-year-old pupils in the fourth grade of Electric Power Primary School, and their school hours were 7:30–11: 00,14: 30–17:30.).

**Valid period**	**Summer** **(June, July, August)**	**Spring (autumn)** **(April, May, September, October)**	**Winter** **(November, December, January, February, March)**
**8:00–9:00**	10%	5%	2%
**9:00–10:00**	14%	7%	5%
**10:00–11:00**	7%	12%	9%
**11:00–12:00**	4%	9%	15%
**12:00–13:00**	2%	5%	8%
**13:00–14:00**	1%	1%	2%
**14:00–15:00**	5%	5%	9%
**15:00–16:00**	6%	9%	13%
**16:00–17:00**	10%	17%	11%
**17:00–18:00**	14%	11%	10%
**18:00–19:00**	18%	9%	8%
**19:00–20:00**	6%	5%	4%
**20:00–21:00**	2%	4%	3%
**21:00–22:00**	1%	1%	1%

**Table 5:** Child behavior scale for 3–5 years old pre-school children. (The following data are the average range of boys and girls).

**Age**	**3 years old**	**4 years old (first half)**	**4 years old (last half)**	**5 years old (first half)**	**5 years old (last half)**
**Squatting height**	81.0–87.0 cm	84.0–84.7 cm	79.3–82.3 cm	73.3–78.7 cm	70.3–66.7 cm
**Squatting latent height**	40.0–40.0 cm	40.0–40.0 cm	40.0–40.0 cm	40.0–40.0 cm	40.0–40.0 cm
**Creeping height**	20.0–20.0 cm	20.0–20.0 cm	20.0–20.0 cm	20.0–20.0 cm	20.0–20.0 cm
**Step distance**	54.3–55.7 cm	60.0–61.0 cm	62.0–63.0 cm	63.7–65.0 cm	64.3–65.3 cm
**Lifting height**	70.0–70.0 cm	66.7–70.0 cm	76.7–80.0 cm	80.0–86.7 cm	110.0–110.0 cm
**Cross height**	40.0–43.3 cm	53.5–53.5 cm	53.5–56.7 cm	53.0–60.0 cm	56.7–56.7 cm
**Crotch height**	36.0–43.7 cm	41.0–44.0 cm	41.7–45.3 cm	47.3–47.7 cm	48.0–50.7 cm
**Jumping down height**	100.0–103.3 cm	106.7–123.3 cm	120.0–126.7 cm	126.7–140.0 cm	150.0–160.0 cm
**Jumping distance**	73.3–73.3 cm	86.7–97.0 cm	101.3–104.0 cm	105.0–110.0 cm	112.3–138.0 cm
**Jumping up height**	–	125.0–128.3 cm	141.7–141.7 cm	143.3–143.3 cm	148.3–150.0 cm
**Height of the middle finger when raising**	119.0–119.0 cm	124.0–124.0 cm	132.0–132.0 cm	135.3–135.3 cm	140.7–140.7 cm
**Rotation height**	–	46.7–50.0 cm	50.0–53.3 cm	50.0–56.7 cm	50.0–50.0 cm

**Table 6:** Comprehensive exclusivity.

**Age level**	**Gender exclusivity**	**Age exclusivity**
**0–2.5**	0.35	0.10
**2.5–3**	0.50	0.20
**3–4**	0.65	0.45
**4–5**	0.75	0.50
**5–8**	0.85	0.75
**8–10**	0.92	0.80
**10–12**	0.95	0.90

**Table 7:** Self – centered.

**Age level**	**Body center**	**Psychological center**
**0–2.5**	0.72	0.55
**2.5–5**	0.80	0.70
**5–6**	0.90	0.94
**6–7**	0.95	0.98
**7–8**	0.92	0.96
**8–10**	0.90	0.88
**10–12**	0.85	0.75

**Table 8:** Five measurement elements of children friendly space.

**Aim level**	**Comprehensive evaluation layer**	**Sub-evaluation layer**	**Detailed evaluation layer**
	**Level 1 indicators**	**Level 2 indicators**	**Level 3 indicators**
**Children friendly space in cold resident areas**	Seasonal circulation 0.14	Climate optimism 0.08	Residential "microclimate" optimization. 0.04
			Landscape conversion rebirth. 0.04
		Spatial adaptability 0.06	Make outdoor space "interior". 0.04
			Movable greenhouse. 0.02
	Accessibility 0.18	Residential planning 0.10	Walking time is short. 0.08
			There is no need to cross the city road. 0.02
		Site accessibility 0.08	Site entrance is eye-catching and convenient. 0.03
			Accessible facilities are complete. 0.05
	Safety 0.38	Social supervision 0.10	The venue is located near the community service center. 0.04
			The vision of whole field is more open. 0.02
			Neighbors are also more receptive to the site. 0.04
		Field sense 0.09	The nearby building shape is easy to identify. 0.02
			The name of the site is simple and easy to remember. 0.03
			The site has a clear boundary and prevents past people, cars, etc. passing through. 0.04
		Field safety 0.19	Pieces, facilities strong, without sharp corners, crowded folder and other security risks. 0.06
			Pavement is done by soft and hard material combination which can play a certain protective effect. 0.05
			Edges of pools, pits and other dangerous areas have protective facilities. 0.05
			No toxic, prickly, contaminated plants. 0.03
	Comfort 0.18	Site space quality 0.07	The overall size of the space is appropriate, and it is not crowded nor open usually. 0.01
			Light and ventilation conditions are both good. 0.01
			There is a suitable shade in summer. 0.01
			There is a large enough pavement for roller skating, baby carriage activities. 0.02
			The rest area is properly separated from the activity area. 0.01
			The site boundary can be combined with greening. 0.01
		Small quality facilities 0.07	There are plenty of comfortable resting locations, with taking into account the needs of adult escorts. 0.01
			Steps, railings and seats can all meet the child scale. 0.02
			There are a small number of facilities, simple and full of comedy, such as sand, pool, terrain climbing frame, slide, etc. 0.03
			The number of toilet and trash are adequate with reasonable location. 0.01
		Green quality 0.04	The main tree species have characteristics, with flowers, leaves and fruit having strong viewing. 0.01
			The seasonal variation of plants is obvious. 0.02
			With interesting popular plants. 0.01
	Versatility 0.12		The venue is suitable for organizing various community activities. 0.04
			The site is suitable for people of all ages. 0.05
			The venue is surrounded by small shops, snack bars, farms and so on. 0.03

**Table 9:** Children' behavioral attributes and spatial form in child-friendly space.

**Children – friendly space type and characteristics**	**The diversity of children's behavior**	**Continuity of child behavior**	**Spontaneity of child behavior**	**Participatory of accompanying staff**
**Green flatness**	Spatial form/positive correlation	Spatial form/positive correlation	Spatial form/positive correlation	Spatial form/positive correlation
**Width of the green space**	Spatial form/positive correlation	Spatial form/positive correlation	Spatial form/positive correlation	Spatial form/positive correlation
**Leisure space**	Space diversification/positive correlation	Seasonal circulation	Space diversification/positive correlation	Space diversification/positive correlation
**Sport space**	Space diversification/positive correlation	Space diversification/positive correlation	Space diversification/positive correlation	Space diversification/positive correlation
**Gray space**	Space diversification/positive correlation	Space diversification/positive correlation	Space diversification/positive correlation	Space diversification/positive correlation
**Thermal conductivity of materials**	Inert soft materials/positive correlation	Inert soft materials/positive correlation	Inert soft materials/positive correlation	Inert soft materials/positive correlation
**Facilities layout distance**	Comfort range/positive correlation	Comfort range/positive correlation	Comfort range/positive correlation	Comfort range/positive correlation
**Facility color mixing degree**	Highly recognizable/positive correlation	Highly recognizable/positive correlation	Highly recognizable/positive correlation	Highly recognizable/positive correlation
**Organism regionalism**	Adaptability/positive correlation	Adaptability/positive correlation	Adaptability/positive correlation	Adaptability/positive correlation
**Car occupancy and walking distance**	Less car use/short walking distance/positive correlation	Less car use/short walking distance/positive correlation	Less car use/short walking distance/positive correlation	Less car use/short walking distance/positive correlation
**Landscape change**	Good circulation/positive correlation	Good circulation/positive correlation	Good circulation/positive correlation	Good circulation/positive correlation

## 2 Experimental design, tools and methods

The dataset was from a field sub-time extended investigation to children (babies, preschool children, school age children) of Hohhot West Inner Mongolia Electric Power Community Residential Area in Inner Mongolia of China that belongs to cold region of ID area according to Chinese design code for buildings. Energy efficiency was done by DSPI model, and measurement elements and spatial form of the child-friendly space was done by Matrix analysis, and resulted from weighting the three-level evaluation of Table 9 [4], [5].

(1) Using the formula of comprehensive energy efficiency to explain the cognition of friendly space in children of different ages: Comprehensive energy efficiency: (Energy Efficient)=S (Season Circulation)*B (Behavior Particularity) *E (Comprehensive exclusivity) *S (Self – centered), and the essence is a DSPI model, so DPSI=Driving-forces (Season driven) -Pressures (Comprehensive exclusivity) -State (Behavior Particularity) -Impacts (Self - centered).
(2) The final analysis is the weighting result of the three-level evaluation: multiplying the matrix of the two-stage comprehensive evaluation vector with the comprehensive weight matrix W, and the three-level comprehensive evaluation vector is obtained. The final different layers are as follows: V={V1 (children's friendliness is high), V2 (child friendliness is higher), V3 (child friendliness is general), V4 (child friendliness is lower), V5 (child friendliness is low)}
